# Gene co-expression network analysis revealed novel biomarkers for ovarian cancer

**DOI:** 10.3389/fgene.2022.971845

**Published:** 2022-10-19

**Authors:** Ceyda Kasavi

**Affiliations:** Department of Bioengineering, Faculty of Engineering, Marmara University, Istanbul, Turkey

**Keywords:** ovarian cancer, transcriptome profiling, differential gene co-expression network, prognostic gene module, biomarkers

## Abstract

Ovarian cancer is the second most common gynecologic cancer and remains the leading cause of death of all gynecologic oncologic disease. Therefore, understanding the molecular mechanisms underlying the disease, and the identification of effective and predictive biomarkers are invaluable for the development of diagnostic and treatment strategies. In the present study, a differential co-expression network analysis was performed *via* meta-analysis of three transcriptome datasets of serous ovarian adenocarcinoma to identify novel candidate biomarker signatures, i.e. genes and miRNAs. We identified 439 common differentially expressed genes (DEGs), and reconstructed differential co-expression networks using common DEGs and considering two conditions, i.e. healthy ovarian surface epithelia samples and serous ovarian adenocarcinoma epithelia samples. The modular analyses of the constructed networks indicated a co-expressed gene module consisting of 17 genes. A total of 11 biomarker candidates were determined through receiver operating characteristic (ROC) curves of gene expression of module genes, and miRNAs targeting these genes were identified. As a result, six genes (*CDT1*, *CNIH4*, *CRLS1*, *LIMCH1*, *POC1A*, and *SNX13*), and two miRNAs (mir-147a, and mir-103a-3p) were suggested as novel candidate prognostic biomarkers for ovarian cancer. Further experimental and clinical validation of the proposed biomarkers could help future development of potential diagnostic and therapeutic innovations in ovarian cancer.

## Introduction

Ovarian cancer is the second most common gynecologic cancer and remains the leading cause of death of all gynecologic oncologic disease (Didžiapetriene et al., 2014; Madden et al., 2014). High-grade serous ovarian cancer, which accounts for 70% of the patients, is the most aggressive type with low survival rate (Coscia et al., 2016) due to late stage diagnosis of the disease (Egan et al., 2011). The mortality rate can be reduced with early diagnosis and personalized treatments. Thereby, the elucidation of molecular mechanisms of serous ovarian cancer pathogenesis and the identification of alternative diagnostic methodologies, particularly effective, reliable and predictive biomarkers still remain a high priority, and would be vital to provide new insights on the disease.

The high-throughput microarray profiling has been extensively used for years to understand the underlying mechanisms of human diseases at molecular level. These studies revealed hundreds of genes that were significantly expressed in complex diseases, including cancers. The identification of transcriptionally dysregulated genes and altered molecular mechanisms in the presence of cancer provides information on tumor’s biological state and the patients’ survival (Otálora-Otálora et al., 2019). Therefore, the integration of these set of genes with biological information at different levels continues to be an effective method to discover molecular targets that may serve as diagnostic and prognostic tools, and help to develop and therapeutic strategies (Gov and Arga, 2017; Venkataramanan and Mathavan, 2020).

A system-based approach is needed to gain a comprehensive view of cancer, and the power of differential co-expression networks was previously shown in cancer research (Gov and Arga, 2017)^.^ The construction and analysis of differential co-expression networks have been widely used to discover gene modules and hub genes in various cancers, including retinoblastoma (Mao et al., 2020), pituitary adenoma (Aydin and Arga, 2019), ovarian (Gov and Arga, 2017; Gov, 2020), lung (Liao et al., 2020)^,^ and cervical cancers (Kori et al., 2019)^.^

The aim of the present study was to provide further insight to the genetic and molecular underpinnings of ovarian cancer pathogenesis and to identify associated molecular signatures. For this purpose, three independent gene expression datasets of serous ovarian adenocarcinoma were comparatively analyzed and 439 common DEGs were identified. The differential co-expression networks were reconstructed in the diseased and healthy states by using common DEGs, and functional modular organizations of the reconstructed networks were examined. A novel gene module consisting of 17 genes that was differentially co-expressed in ovarian cancer, but not preserved in healthy state, was identified. ROC curve analysis indicated high diagnostic performances of 11 module genes that might serve as potential biomarkers. Moreover, miRNAs targeting these 11 genes were screened to identify regulatory signatures of the disease. As a result, six genes (*CDT1*, *CNIH4*, *CRLS1*, *LIMCH1*, *POC1A*, and *SNX13*) and two miRNAs (mir-147a, and mir-103a-3p) were determined as novel biomarker candidates and putative therapeutic targets that would be invaluable for future studies.

## Materials and methods

### Gene expression data collection

The gene expression datasets associated with serous ovarian adenocarcinoma from three independent studies [GSE10971 (Tone et al., 2011), GSE14407 (Bowen et al., 2009), and GSE18520 (Mok et al., 2009)] were obtained from Gene Expression Omnibus (GEO) database (Table 1). The studies that used the platform GPL570 (Affymetrix Human Genome U133 Plus 2.0 arrays) were selected to avoid altered gene expressions due to microarray differences. GSE10971 contained gene expression profiles of laser capture micro-dissected non-malignant distal fallopian tube epithelium from 12 BRCA1/2-mutation carriers and 12 control women during the luteal and follicular phase, and 13 high grade tubal and ovarian serous carcinomas. Samples of BRCA1/2-mutation carriers were excluded from this dataset. GSE14407 was consisted of 12 healthy ovarian surface epithelia samples and 12 laser-capture micro-dissected papillary serous ovarian adenocarcinoma epithelia samples. GSE18520 contained 53 micro-dissected late stage, high-grade primary papillary serous ovarian adenocarcinoma and 10 normal ovarian surface epithelia samples.

**TABLE 1:** Gene expression data sets used in this study.

GEO	Control	Disease	Array	References
GSE10971	12	13	GPL570	Tone et al. (2011)
GSE14407	12	12	GPL570	Bowen et al. (2009)
GSE18520	10	53	GPL570	Mok et al. (2009)

### Identification of differentially expressed genes

DEGs were identified using the GEO2R tool (http://www.ncbi.nlm.nih.gov/geo/geo2r/), which uses LIMMA (Smyth, 2004) as the statistical test. Benjamini–Hochberg’s method was used to control the false discovery rate (FDR). The statistical significance and expression patterns (up- or down-regulation) of DEGs were determined by an adjusted *p*-value threshold and fold-change (FC), respectively. An adjusted *p*-value threshold of 0.01 and FC cut-off of two were used to identify significant DEGs.

### Pathway enrichment and gene-disease association analyses

Pathway enrichment analyses were carried out by Metascape (Zhou et al., 2019) (https://metascape.org/gp/index.html#/main/step1) to identify significant KEGG (Kanehisa and Goto, 2000) pathways associated with DEGs. The *p*-values were obtained *via* hypergeometric test and corrected using Benjamini–Hochberg algorithm. An adjusted *p*-value threshold of 0.05 was used to identify the statistical significance of the identified pathways. Significant disease associations of DEGs were determined using DisGeNet database (Piñero et al., 2020) *via* R (R Core Team, 2020) with a *p*-value threshold of 0.05.

### Construction of differential gene co-expression networks and modular analysis

The differential co-expression networks were constructed for the diseased and healthy states separately considering common DEGs in three datasets. Two expression datasets were obtained by separating the expression values of common DEGs in healthy and diseased states. The mean expression values were used for repetitive DEGs. The healthy and disease datasets were composed of 34 and 78 samples, respectively. Both datasets were normalized *via* quantile normalization performed by RMA (Bolstad et al., 2003). The correlations between the expression profiles of common DEGs were determined by Pearson correlation coefficients (PCCs) computed between each gene pair in both datasets, and the statistical significance of pairwise correlations were determined *via* asymptotic *p*-values approximated by using the t-distribution. An absolute PCC cut-off of 0.8 and an asymptotic *p*-value threshold of 0.05 were maintained to identify the statistically significant correlations. PCC >0.8 and PCC < −0.8 were defined as positive and negative correlations among gene pairs, respectively. The differential gene co-expression networks, i.e., disease network (OC), and control network (non-OC), were constructed around the significantly co-expressed gene pairs in the diseased, and healthy states, respectively.

Constructed networks were visualized *via* Cytoscape (v3.7.2) (Shannon et al., 2003)^,^ and CytoHubba (Chin et al., 2014) was used to compute node scores. Genes were ranked based on degree and 5% of the genes with the highest degree scores were determined as hub genes.

Highly connected subgroups within the gene co-expression networks were identified *via* MCODE (Bader and Hogue, 2003) plug-in of Cytoscape. In MCODE, network scores were computed by excluding the loops. Modules were identified setting the degree threshold, node score threshold, K-core threshold, and maximum depth to 2, 0.2, 2 and 100, respectively. The fluff parameter was turned off and the hair-cut parameter was turned on. The most densely connected regions (the top-scoring modules) of both networks were taken into consideration for further analysis.

### Diagnostic performances of module genes

The gene expression profiles of OC module (OCM) genes were extracted from the three datasets, and receiver operating characteristic (ROC) curves were plotted. Area under the curve (AUC) representing sensitivity (the proportion of positive test results in patients) and specificity (the proportion of negative test results in healthy individuals) was used to evaluate the diagnostic performance of the genes.

### Nucleosome organization analysis

The nucleosome positioning patterns of ovarian cancer associated co-expressed genes were screened using NucMap database (Zhao et al., 2019), which contains 798 experimental data from 477 samples across 15 species. The enrichment analyses of nucleosome occupancy were carried out using normalized raw reads across four different cell lines representing chronic myelogenous leukemia (K-562), skin melanoma (COLO829), cervical carcinoma (HeLa), and breast adenocarcinoma (MCF-7). The nucleosome enrichment around transcription start sites (TSSs) were visualized.

### Construction of miRNA-target gene regulatory network

The miRNA-target gene regulatory network was constructed by mapping prognostic module genes to the respective miRNAs. The miRNet 2.0 database (Chang et al., 2020) was used to identify miRNA-gene interactions with a degree cut-off of 3, and to visualize the constructed network.

## Results

### Differentially expressed genes in ovarian cancer

**FIGURE 1:**
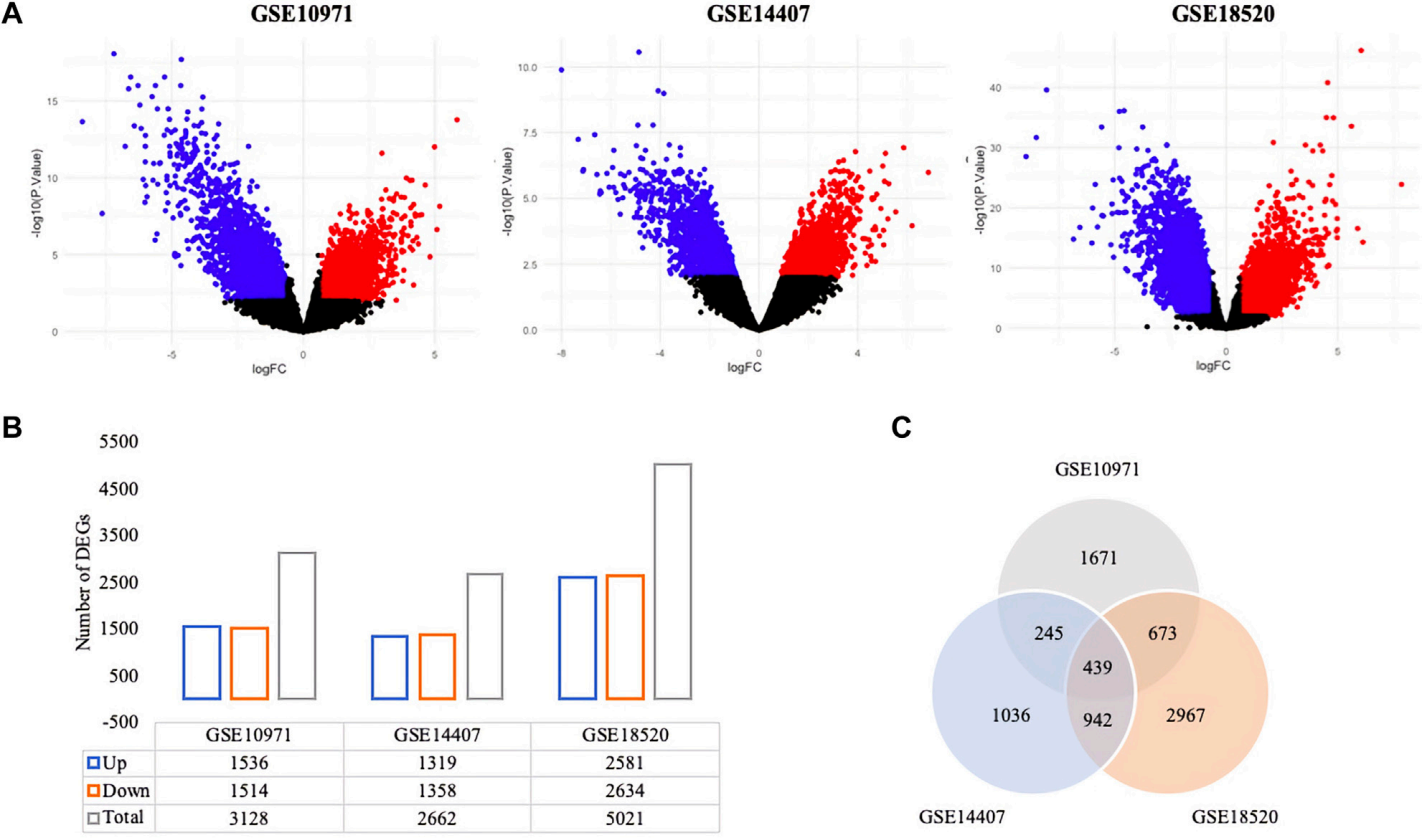
Differentially expressed genes (DEGs) in ovarian cancer. **(A)** The volcano plots of gene expression datasets; red and blue dots represent the up- and down-regulated DEGs at an adjusted *p*-value threshold of 0.01, respectively. **(B)** The distribution of up- and down-regulated DEGs (adjusted *p*-value < 0.01). **(C)** Venn diagram representation of DEGs across gene expression datasets.

**FIGURE 2:**
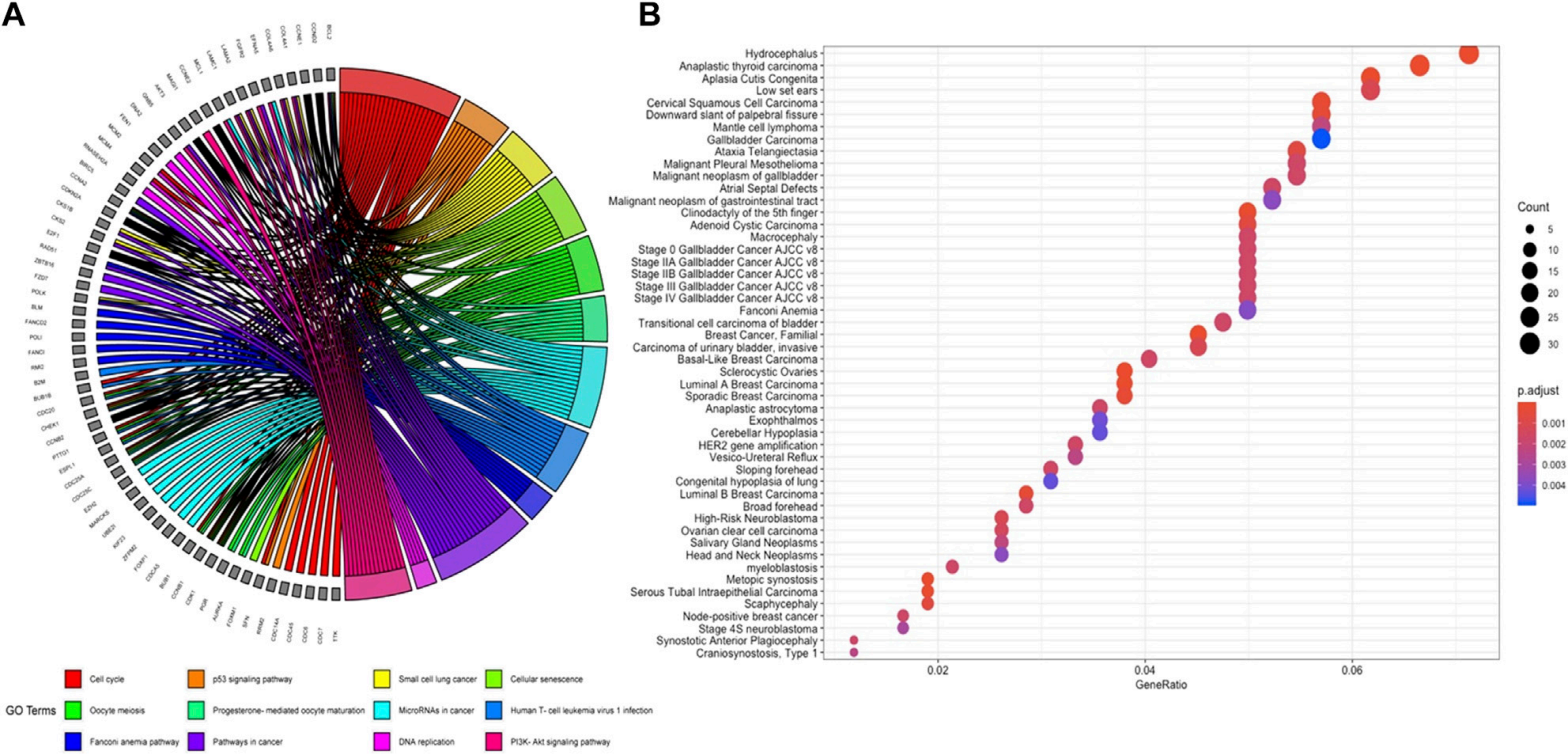
**(A)** GOChord plot of significantly enriched KEGG pathways. **(B)** Significantly associated diseases of common DEGs in ovarian cancer.

Genome-wide response of ovarian epithelia cells to the presence of serous ovarian adenocarcinoma at the transcriptional level revealed numerous genes that were significantly and differentially expressed. The individual analysis of gene expression datasets resulted in the identification of 4,263 up-regulated and 4,313 down-regulated DEGs (Figure 1A). A total of 1,536 up- and 1,514 down-regulated genes in GSE10971, 1,319 up- and 1,358 down-regulated genes in GSE14407, and 2,581 up- and 2,634 down-regulated genes in GSE18520 were determined (Figure 1B). Although the numbers of DEGs among datasets varied, comparative analysis revealed 439 common DEGs (Figure 1C). The pathway enrichment analyses of common DEGs revealed cell cycle, oocyte meiosis, Fanconi anemia pathway, and several cancer-associated pathways as significant pathways (Figure 2A). Moreover, the gene-disease association analyses indicated a significant relation between common DEGs and several cancers (Figure 2B).

### Differential co-expression networks in ovarian cancer

**FIGURE 3:**
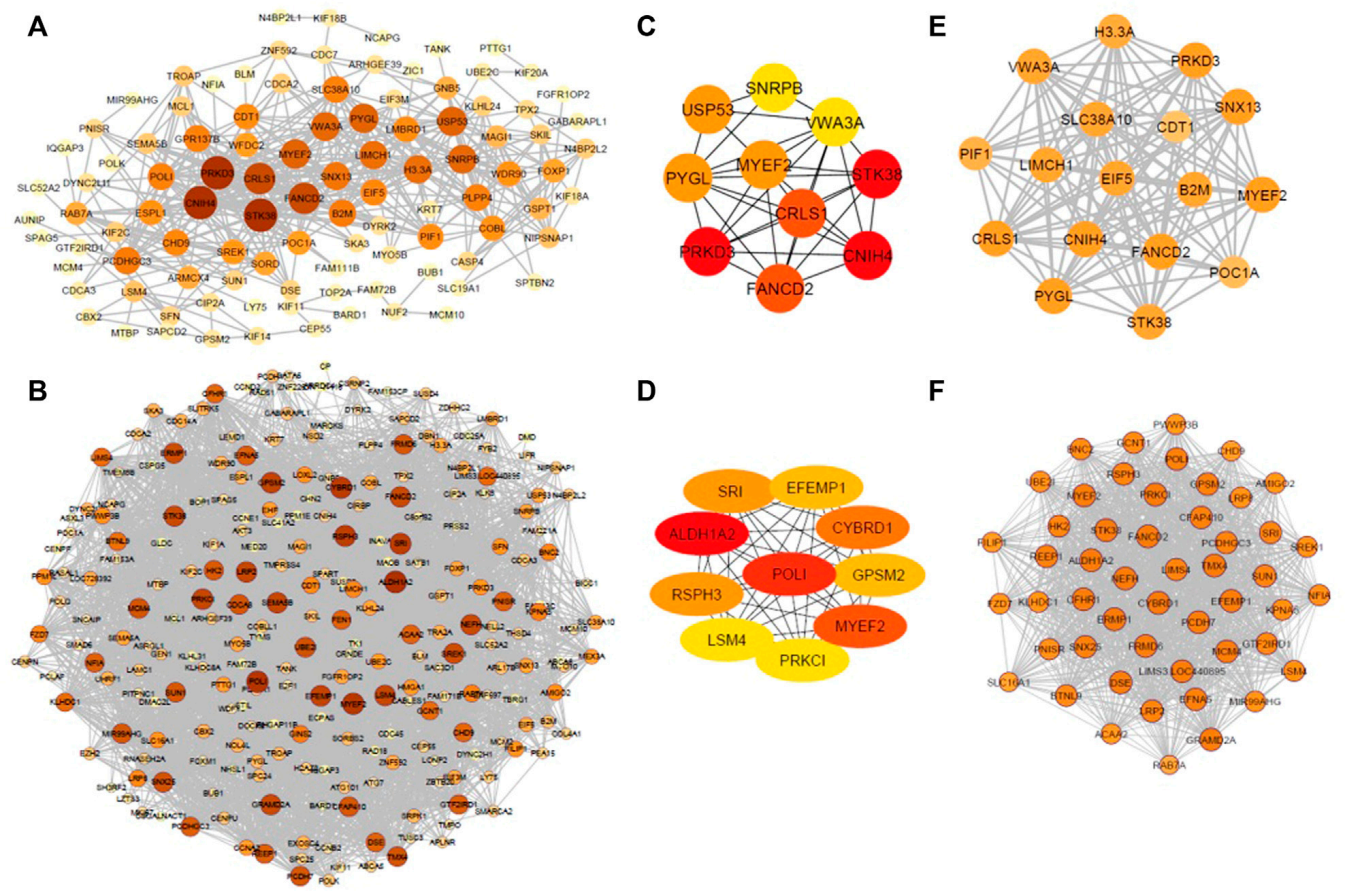
**(A)** OC, constructed around the significantly co-expressed gene pairs in the diseased state. **(B)** non-OC, constructed around the significantly co-expressed gene pairs in healthy state. **(C)** Hub genes of OC. **(D)** Hub genes of non-OC. **(E)** Top-scoring module of OC module (OCM). **(F)** Top-scoring module of non-OC. Size and colors of nodes were determined according to node degrees.

The co-expression patterns of common DEGs were analyzed in the diseased state and healthy state separately to construct diseased and control co-expression networks, respectively. The employment of PCC’s resulted in a total of 3,375 significant correlations among 271 genes. OC, constructed around the significantly co-expressed gene pairs in the diseased state, contained 522 associations among 113 DEGs (Figure 3A), and non-OC, constructed around the significantly co-expressed gene pairs in healthy state, consisted of 3,044 associations among 250 DEGs (Figure 3B). The topological analysis indicated that both networks had the same density (%10), although the number of genes showing significant pairwise correlations was 2.2-fold lower in OC when compared to non-OC.

A total of 8 and 16 genes were determined to be hub genes in OC, and non-OC, respectively. *CNIH4*, *PRKD3*, *CRLS1*, *USP53*, and *PYGL* were found to be specific to OC. *ALDH1A2*, *POLI*, *CYBRD1*, *RSPH3*, *SRI*, *EFEMP1*, *GPSM2*, *LSM4*, *PRKCI*, *SEMA5B*, *LRP2*, *NEFH*, *GRAMD2A* were identified as specific hub genes for non-OC. Comparative analysis revealed *FANCD2*, *MYEF2*, and *STK38* as the mutual hub genes (Figures 3C,D).

### Co-expressed gene module of ovarian cancer

**FIGURE 4:**
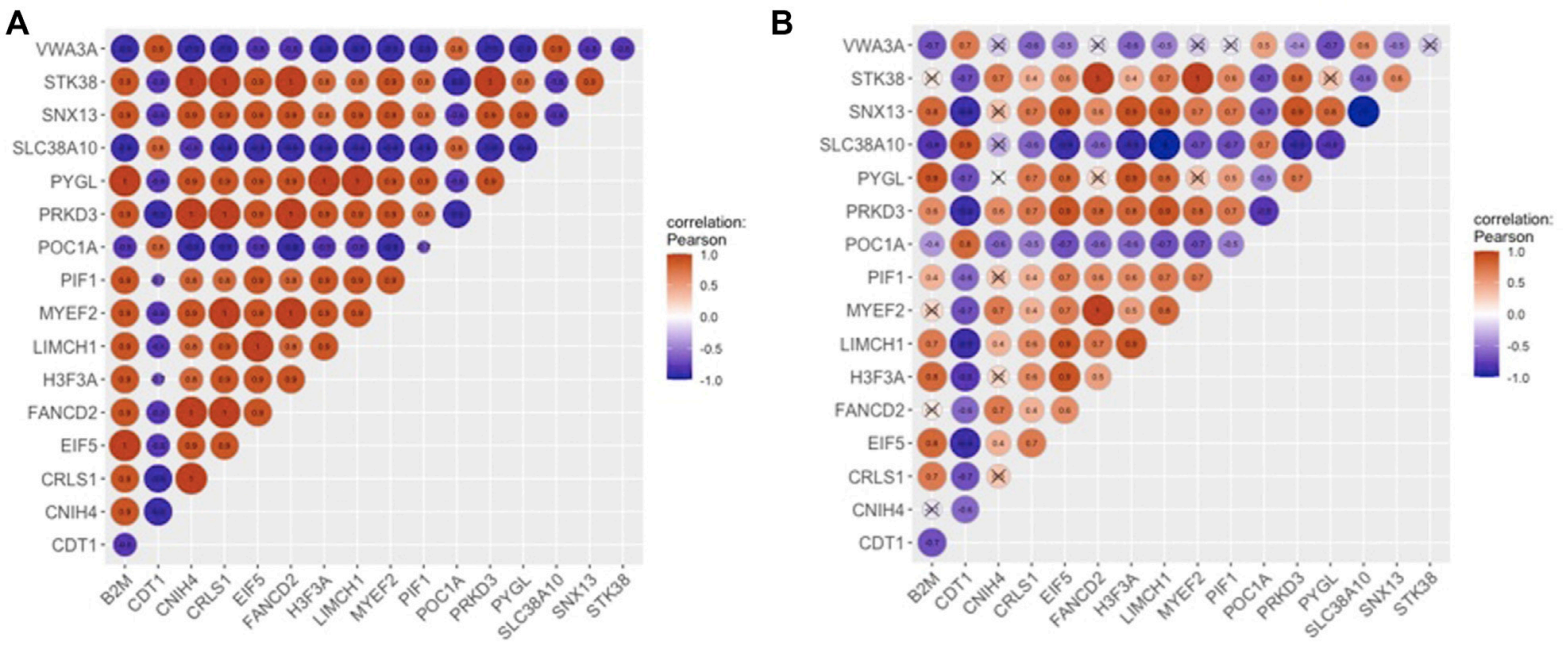
Correlations of OCM **(A)** in the diseased state, and **(B)** in healthy state. The areas of circles show the absolute values of corresponding PCC’s.

The most densely connected regions of OC and non-OC were identified by MCODE. OCM contained 125 interactions between 17 genes (Figure 3E), whereas non-OC module (non-OCM) consisted of 1,149 interactions between 51 genes (Figure 3F). Comparative analysis of the modules indicated that only three genes (*FANCD2*, *MYEF2*, and *STK38*), which were also mutual hub genes of OC and non-OC, were found to be common in OCM and non-OCM. To assess the prognostic capability of OCM genes in ovarian cancer, we evaluated whether OCM was preserved in healthy state. The preservation analysis was carried out by comparing the significant correlations among OCM genes with their correlations in healthy samples. PCC values that show the correlations between OCM genes in diseased and healthy samples are presented in Supplementary Table S1. Only 33 associations among 13 OCM genes were found to be significant among healthy samples (Figure 4). The module density, which was 0.919 in the diseased state, was found to be 2.2-fold lower (0.423) in healthy state. Since OCM genes were highly correlated in the diseased state, but not in healthy state, this module was considered as a prognostic module in ovarian cancer. *B2M*, *CDT1*, *CNIH4*, *CRLS1*, *EIF5*, *FANCD2*, *H3-3A*, *LIMCH1*, *MYEF2*, *PIF1*, *POC1A*, *PRKD3*, *PYGL*, *SLC38A10*, *SNX13*, *STK38*, and *VWA3A* were the correlated DEGs in OCM. Interestingly seven genes had the highest degree that corresponds to interactions with 16 genes in the module. These genes were *CNIH4*, *CRLS1*, *FANCD2*, *MYEF2*, *PRKD3*, *PYGL*, and *SNX13*, and they were positively correlated with each other, while all of them were negatively correlated with four genes, including *CDT1*, *POC1A*, *SLC38A10*, and *VWA3A*. *CDT1* had the least number of interacting partners (a degree of 10) in the module.

The descriptions and functions of OCM genes are presented in Table 2. Functional enrichment analysis of OCM showed that the prognostic module genes were not significantly associated with any KEGG pathways, but they were significantly enriched with regulation of chromosome organization, negative regulation of cellular component organization, and DNA metabolic process GO biological processes.

**TABLE 2:** The descriptions of genes in OCM.

Gene	Description	Function
*B2M*	beta-2-microglobulin	It encodes a serum protein found in association with the major histocompatibility complex (MHC) class I heavy chain on the surface of nearly all nucleated cells
*CDT1*	chromatin licensing and DNA replication factor 1	It is required for both DNA replication and mitosis
*CNIH4*	cornichon family AMPA receptor auxiliary protein 4	It is involved in G protein-coupled receptors (GPCRs) trafficking from the endoplasmic reticulum to the cell surface
*CRLS1*	cardiolipin synthase 1	It catalyzes the synthesis of cardiolipin which is a key phospholipid in mitochondrial membranes and plays important roles in maintaining the functional integrity and dynamics of mitochondria under both optimal and stress conditions
*EIF5*	eukaryotic translation initiation factor 5	The related pathways of this gene are activation of the mRNA upon binding of the cap-binding complex and eIFs, and subsequent binding to 43S and viral mRNA translation
*FANCD2*	FA complementation group D2	It is required for maintenance of chromosomal stability, promotes accurate and efficient pairing of homologs during meiosis, and involved in the repair of DNA double-strand breaks. It is associated with Fanconi anemia
*H3-3A*	H3.3 histone A	The related pathways of this gene are transcriptional misregulation in cancer and signaling by GPCR
*LIMCH1*	LIM and calponin homology domains 1	It positively regulates actin stress fibers assembly and stabilizes focal adhesions, and therefore negatively regulates cell spreading and cell migration
*MYEF2*	myelin expression factor 2	It is a transcriptional repressor of the gene encoding myelin basic protein
*PIF1*	PIF1 5′-to-3′ DNA helicase	It is required for the maintenance of both mitochondrial and nuclear genome stability. It is associated with diseases including uterine adnexa cancer and Fanconi anemia
*POC1A*	POC1 centriolar protein A	It plays an important role in basal body and cilia formation
*PRKD3*	protein kinase D3	It encodes a protein that has several functions including negative regulation of human airway epithelial barrier formation, growth regulation of breast and prostate cancer cells, and vesicle trafficking
*PYGL*	glycogen phosphorylase L	It encodes a glycogen phosphorylase that is required to release glucose-1-phosphate from liver glycogen stores
*SLC38A10*	solute carrier family 38 member 10	It is involved in amino acid transmembrane transporter activity
*SNX13*	sorting nexin 13	It encodes a protein that is the regulator of G protein signaling (RGS) family and may link heterotrimeric G protein signaling and vesicular trafficking
*STK38*	serine/threonine kinase 38	It has functions in cell cycle and apoptosis
*VWA3A*	von Willebrand factor A domain containing 3A	It is a protein coding gene

**FIGURE 5:**
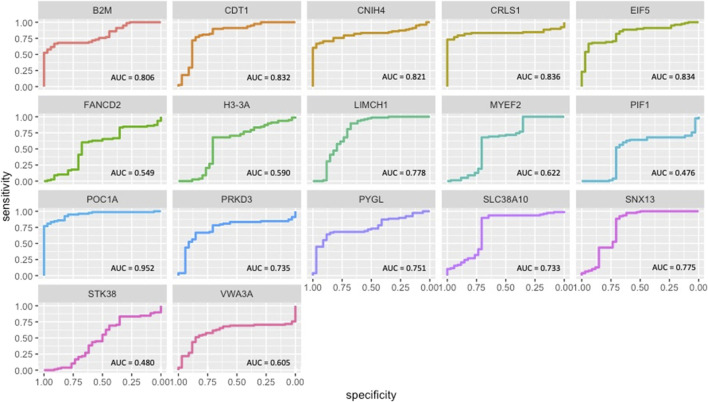
ROC curves of OCM genes.

ROC curve analysis indicated that 11 module genes (*B2M*, *CDT1*, *CNIH4*, *CRLS1*, *EIF5*, *LIMCH1*, *POC1A*, *PRKD3*, *PYGL*, *SLC38A10*, and *SNX13*) showed high performances (AUC ≥0.7) in discriminating diseased patients from healthy controls (Figure 5).

### Establishment of miRNA-target gene regulatory network

**FIGURE 6:**
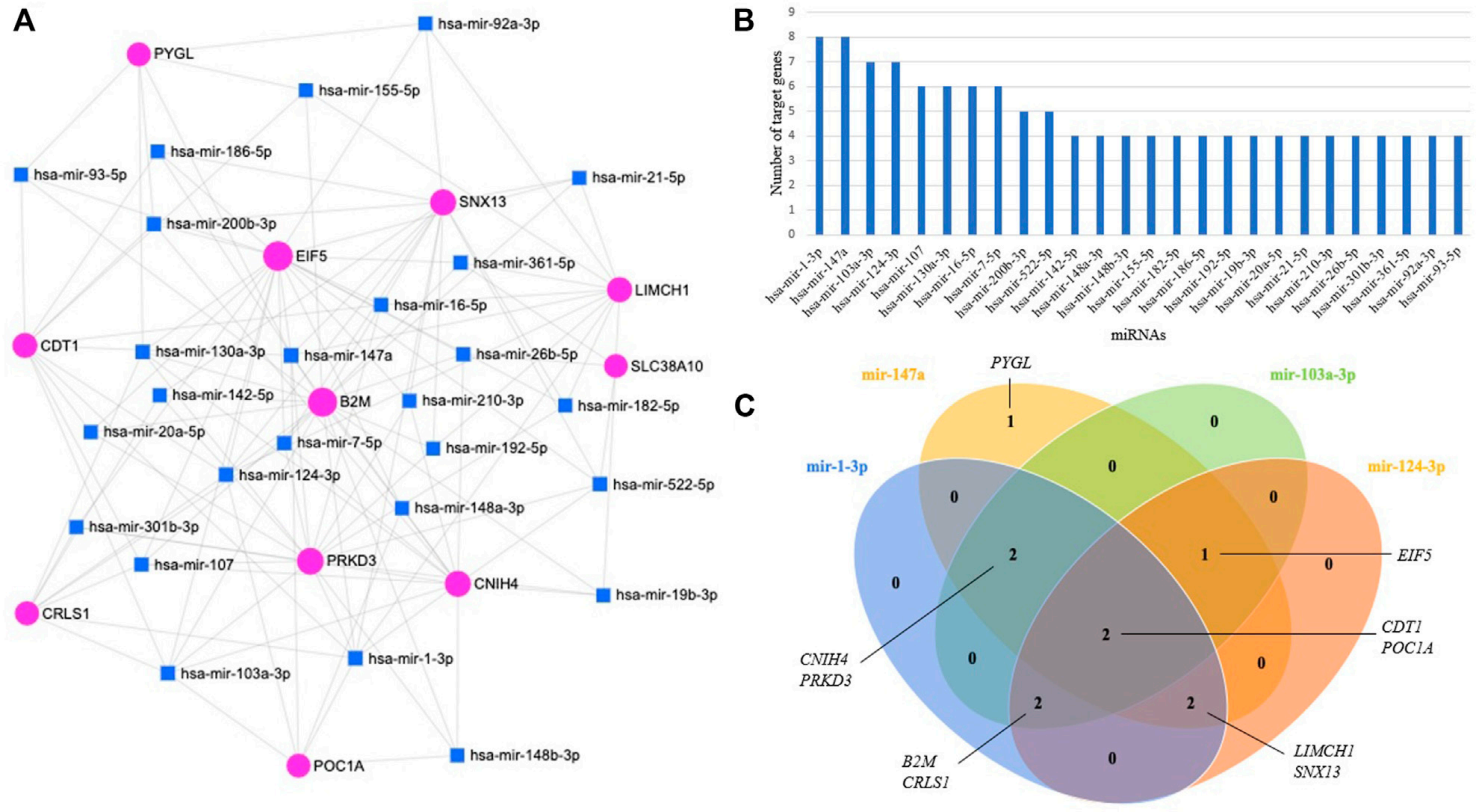
**(A)** miRNA-target gene regulatory network; the blue squares, and pink circles represent miRNAs, and genes, respectively. **(B)** Bar plot representing the distribution of miRNAs regulating genes with high diagnostic performance. **(C)** Venn diagram representing genes regulated by top four miRNAs.

miRNAs have major roles in the regulation of gene expression. A total of 26 miRNAs targeting 11 prognostic module genes were identified using miRNet 2.0. Only miRNAs having a degree higher than three were presented (Figure 6A). When miRNA-target gene interactions were analyzed, miR-1-3p, miR-147a, miR-103a-3p, and miR-124–3p came into prominence by regulating at least seven genes (Figure 6B). Two genes, *CDT1* and *POC1A*, were found to be commonly regulated by the top four miRNAs (Figure 6C). The analysis revealed that the majority (91%) of prognostic module genes were regulated by miR-1-3p, and miR-147a.

### Nucleosome positioning at transcription start sites of ovarian cancer associated genes

**FIGURE 7:**
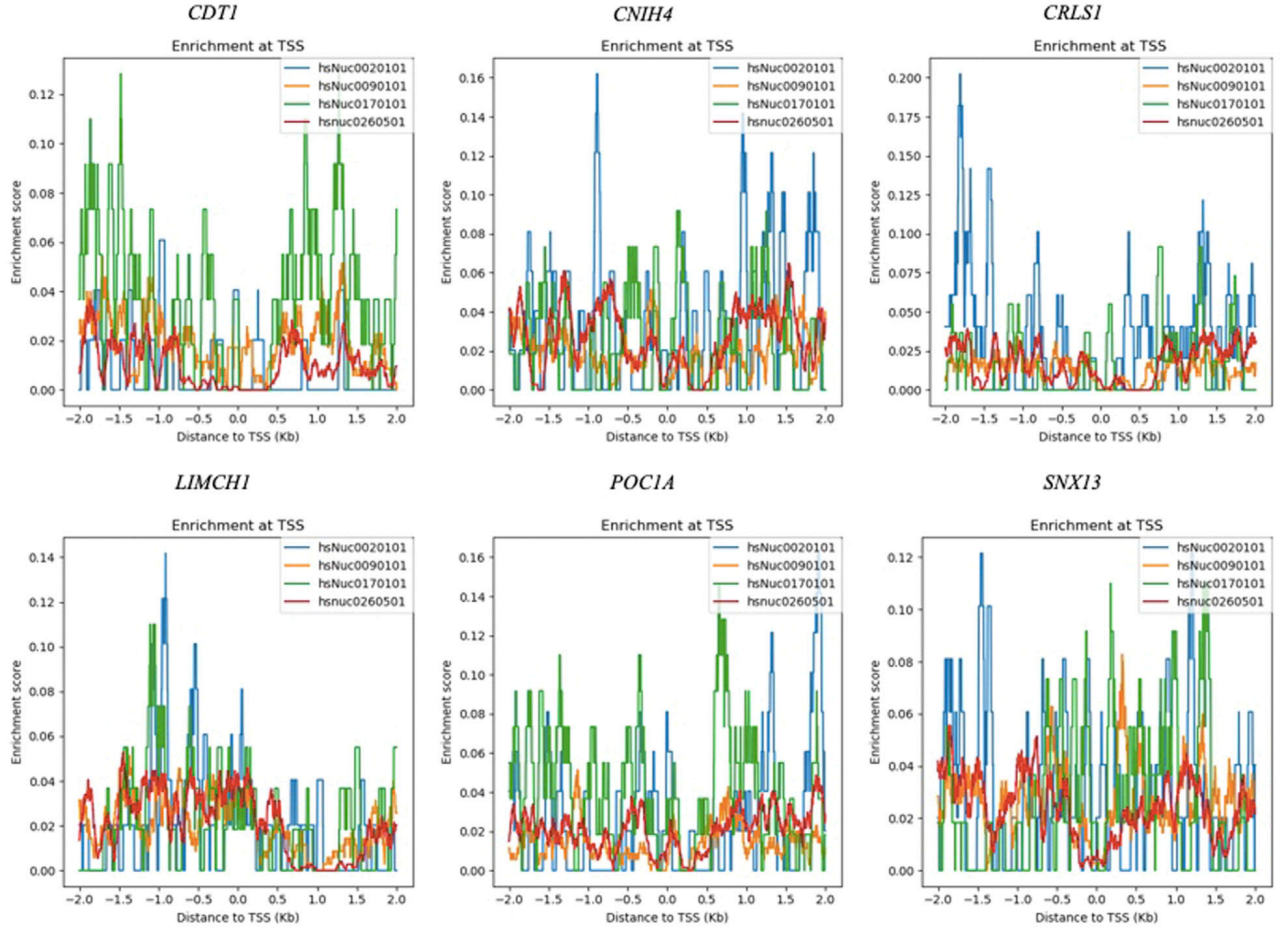
Nucleosome enrichment maps of novel candidate genes at K-562 (hsNuc0260501), COLO829 (hsNuc0020101), HeLa (hsNuc0170101), and MCF-7 (hsNuc0090101) cells.

The prognostic module, OCM, contained genes that were already reported to be associated with ovarian cancer together with novel candidates, such as *CDT1*, *CNIH4*, *CRLS1*, *LIMCH1*, *POC1A*, and *SNX13*. The nucleosome enrichment analyses of novel candidate genes were carried out across samples including breast adenocarcinoma, chronic myelogenous leukemia, skin melanoma, and cervical carcinoma cell lines to enlighten the nucleosome distribution around TSSs. Although the nucleosome distribution around TSSs did not show a common pattern across samples, the enrichment scores were found to be higher in skin melanoma, and cervical cancer cells when compared to chronic myelogenous leukemia, and breast adenocarcinoma cells for all genes (Figure 7). Low nucleosome occupancy that corresponds to large nucleosome-free regions were detected at TSSs of *CDT1*, *CRLS1*, and *SNX13* in chronic myelogenous leukemia cells, and at TSSs of *POC1A*, and *CRLS1*in breast adenocarcinoma cells. In all samples the nucleosomes were found to be enriched at the downstream of TSS for *CRLS1* (Figure 7).

## Discussion

Ovarian cancer is the leading cause of cancer associated deaths among all gynecological cancers (Bowen et al., 2009). Due to its lethality, the identification of predictive and effective biomarkers with high prognosis is still an issue. The interpretation of omics data is needed to enlighten the complex molecular mechanisms behind disease pathogenesis. Microarray technology has been extensively used in clinical applications and integrative analysis of multiple gene expression datasets has been performed for the identification of significant biomarkers (Yang et al., 2019)^.^

Differential co-expression network analysis can serve as a tool for the identification of correlated molecular targets as potential predictive markers. The elucidation of the alterations in the co-expression patterns of genes in the diseased, and healthy states not only increase our understanding in the pathogenesis of the disease, but also provide valuable information for biomarker discovery. Therefore, in this study, a differential co-expression network analysis was performed to determine ovarian cancer genes and their expression patterns. Comparative analysis of the co-expression networks in the diseased, and healthy states revealed a novel co-expressed gene module that may be regarded as molecular targets offering future development of predictive prognostic biomarkers, and therapeutic strategies in ovarian cancer.

Specifically, we carried out a meta-analysis of three transcriptome datasets, and identified 439 common DEGs. The pairwise correlations amongst common DEGs were determined and the significantly correlated gene pairs were used to construct the differential co-expression networks in the diseased, and healthy states (OC and non-OC, respectively) separately. The modular analyses of the resultant networks exhibited densely connected and functionally related gene groups in both conditions and the comparative analyses of OC and non-OC modules resulted in a prognostic module, namely OCM, composed of 17 genes. The connectedness of the OCM genes was found to be significantly lower in healthy state, which inferred an increased active communication among these genes during the occurrence and/or the development of the disease. Moreover, 11 OCM genes (*B2M*, *CDT1*, *CNIH4*, *CRLS1*, *EIF5*, *LIMCH1*, *POC1A*, *PRKD3*, *PYGL*, *SLC38A10*, and *SNX13*) showed high diagnostic performance. Hence this set of genes were considered as potential prognostic markers.

The candidate prognostic gene set contained genes that were already shown to be associated with ovarian cancer together with novel candidates. *B2M* is the regulator of immune system and its significantly higher expression was observed in the presence of ovarian tumor. *In vitro* analysis using SKOV3 ovarian cancer cell line emphasized that *B2M* knock-down resulted in a decreased cell proliferation, migration and invasion, and an antibody against *B2M* induced apoptosis. Moreover, it was reported that *B2M* was regulated by TGF-β signaling pathway. (Sun et al., 2016). The protein expression of EIF5A1 was induced in epithelial ovarian cancer and high levels of this protein predicted poor survival (Zhang et al., 2018). *PRKD3* belongs to the family of protein kinases, whose members include many oncogenes and growth factor receptors. Some protein kinases were involved in the pathogenesis and progression of breast cancer, and *PRKD3* was linked to prostate cancer, however, very rare mutations of *PRKD3* was observed in ovarian cancer (Liu et al., 2015). On the other hand, the analysis of phosphoproteomic data indicated that the protein abundance of PRKD3 was correlated with poor overall survival of patients with high-grade serous ovarian cancer (Tong et al., 2019)^.^ The proteomic analysis exhibited the expression of PYGL, the liver isoform of glycogen phosphorylase, in SKOV3ip1 and TYK-nu ovarian cancer cell lines (Coscia et al., 2016; Curtis et al., 2019). In another study, it was found that *PYGL* was up-regulated in cisplatin-resistant human ovarian carcinoma cell line SKOV3/DDP when compared to cisplatin-sensitive human ovarian carcinoma cell line SKOV3 (Xu et al., 2018)^.^ A study investigating the role of solute carrier family of membrane transporters in ovarian cancer revealed the down-regulation of *SLC38A10* in ovarian cancer tissues when compared to normal ovarian tissues (Sun et al., 2020).

Among candidate gene set, *POC1A* and *SNX13* have limited information on their association with ovarian cancer. *POC1A* that takes part in the formation of centrioles, and plays a role in ciliogenesis, was found to be correlated with lymphatic metastasis in gastric cancer (Lu et al., 2020). *POC1A* was identified as a hub gene in high grade serous ovarian cancer through network analysis (Wu et al., 2020). In one study, *SNX13* was linked to resistance to chemotherapy in ovarian cancer (Lloyd et al., 2015) In addition, *SNX13* was reported to be related to colorectal cancer migration, invasion, and metastasis (Du et al., 2020). Furthermore, *LIMCH1*, *CRLS1*, *CDT1*, and *CNIH4* were found to be associated with various cancer types, but their roles in ovarian cancer have not been identified yet. *LIMCH1* that regulates non-muscle myosin-II activity and suppresses cell migration, (Lin et al., 2017), was previously proposed in lung (Zhang et al., 2019), breast (Bersini et al., 2020), and endometrial cancers (Bell, 2014). Moreover, *LIMCH1* was identified as a gene signature with high prognostic power in aggressive cervical cancer (Halle et al., 2021). Multiple LIM domain genes, such as *LMX1B* and *PDLIM4* contributed to the tumorigenesis of ovarian cancer. *LMX1B* (LIM homeobox transcription factor 1 beta) was reported as an oncogene in human ovarian cancer based on the correlation between its increased expressions with poor outcome (He et al., 2014). The down-regulation of *PDLIM4* (PDZ and LIM domain 4) was shown to be correlated with short overall survival of ovarian cancer patients (Jia et al., 2019). However, the association of *LIMCH1* with ovarian cancer has not been identified yet.

*CRLS1*, is responsible for cardiolipin production. Reduced expression of cardiolipin was associated with increased apoptosis (Egan et al., 2011). A study of cardiolipin metabolism suggested the tumor suppressor activity of *CRLS1* in non-small cell lung cancer, and hepatocellular carcinoma (Ahmadpour et al., 2020). Moreover, increased cardiolipin levels were observed in cells expressing cytoglobin involved in cancer progression (Thorne et al., 2021). However, there is a limited information with regard to its involvement in ovarian cancer.

Cell cycle regulation and DNA damage response contribute to genome stability and integrity. *CDT1* is a central cell cycle regulator, and links the cell cycle regulation and DNA damage response pathways (Kanellou et al., 2020). *CDT1* might be a novel target in ovarian cancer due to its relationship with other cancers, and with the proteins that were reported to be involved in the progression of the disease. *CDT1* was proposed as a potential biomarker in hepatocellular carcinoma through the analysis of gene expression profiles, and its role was validated *in vitro* (Cai et al., 2021). The high expression of *CRL4*, encoding an E3 ligase, in human ovarian cancer tissues was shown and the repression of *CRL4* mimicked the genotoxic effects of an anticancer agent MLN4924. The regulation of cell proliferation by CLR4^CDT2^ ubiquitin ligase was due to the degradation of its important substrates, such as *CDT1*, *p21*, and *SET8*. The depletion of CLR4^CDT2^ led to the accumulation of *CDT1* and MLN4924 induced apoptotic death was partially rescued by the depletion of *CDT1*, indicating a role to *CDT1* as a possible target in ovarian cancer development and treatment (Pan et al., 2013; Wu et al., 2021).

*CNIH4*, which was also a hub gene that played a pivotal role in the flow of information within OC network, has no reported association with ovarian cancer. *CNIH4* was found to be associated with colon cancer (Mishra et al., 2019) and hepatocellular carcinoma (Wang H. et al., 2021). *CNIH4* interacts with GPCRs (Sauvageau et al., 2014)^.^ A number of GPCRs have been implicated in cancer progression. They are not only contributed to tumor cell growth, but also have several functions in metastasis. Despite the identification of a number of GPCRs with altered expressions due to tumor development, only a few drugs targeting GPCRs were successful. One of the strategy in drug discovery is to target the specific interactions between GPCRs and their ligands (Arakaki et al., 2018; Usman et al., 2020)^.^ Therefore, *CNIH4* might be suggested as a novel therapeutic target due to its topological importance in the network and its role in GPCR trafficking. Although *LIMCH1*, *CRLS1*, *CDT1*, *CNIH4* have been associated with various cancers, their relationship with ovarian cancer has been proposed in this study for the first time to the best of our knowledge.

Nucleosome organization plays an important role in regulatory activities that determines the biological function (Beshnova et al., 2014). The distribution of nucleosomes and the presence of nucleosome-free regions at TSS is strongly associated with transcription initiation. Larger nucleosome-free regions at TSS was reported for highly expressed genes (Chen et al., 2021). No common pattern for nucleosome distribution around TSSs could be observed across samples through the analysis of nucleosome enrichment maps of *CDT1*, *CNIH4*, *CRLS1*, *LIMCH1*, *POC1A*, and *SNX13*. However, the nucleosome occupancy of *CRLS1* at the TSS was lower when compared to other genes for all samples. Further experimental analysis should be carried out to uncover nucleosome organization around TSSs of these highly co-expressed genes in ovarian cancer samples.

The alterations in the expressions of miRNAs have been reported to be associated with invasion and metastasis of ovarian cancer (Chen et al., 2019). Here, four miRNAs (miR-1-3p, miR-147a, miR-103a-3p, and miR-124–3p) came to the fore. Of the significant miRNAs, miR-1-3p showed a decreased expression in ovarian cancer tissues and cell lines, and suppressed the growth and metastasis of ovarian cancer cells (Zhu et al., 2020). miR-124–3p regulates tumorigenesis and progression in several cancers, including ovarian, breast, gastric, bladder cancers, leukemia, and hepatocellular carcinoma (Li et al., 2021). Although previous studies showed the association of miR-147a and miR-103a-3p with various cancers, their roles in ovarian cancer have not been identified yet. miR-147 plays important roles in cell proliferation, apoptosis, and migration, and its expression was reported to be significantly altered in various cancers or carcinomas, including gastric cancer, colon cancer, and hepatocellular carcinoma (Lin and Hu, 2021). miR-147a is involved in the regulation of cancer malignancy (Lee et al., 2021), and was previously proposed as a potential prognostic factor for non-small cell lung cancer (Lu and Luan, 2019). miR-103a-3p has been reported to be associated with cisplatin resistance in non-small cell lung cancer (Wang Z. et al., 2021), and docetaxel resistance in prostate cancer (Yi et al., 2022). miR-103a was also reported as a new regulator of Wnt signaling pathway in colorectal carcinoma (Fasihi et al., 2018).

The current lack of robust diagnostic and prognostic biomarkers owing to the complex nature of the disease, together with the high mortality rate, necessitate new approaches for the discovery of novel biomarkers. Within the framework of this study, the transcriptional response of ovarian epithelia samples to the presence of serous ovarian adenocarcinoma was analyzed. We presented a novel prognostic gene module that was differentially co-expressed in ovarian cancer. Together with the genes that were already known to be associated with the disease (i.e., *B2M*, *EIF5*, *PRKD3*, *PYGL*, *SLC38A10*), some novel candidates were identified. Our findings suggested six genes (*CDT1*, *CNIH4*, *CRLS1*, *LIMCH1*, *POC1A*, and *SNX13*), and two miRNAs (mir-147a, and mir-103a-3p) to serve as novel potential biomarkers that permit future development of diagnostic and therapeutic innovations. However, further experimental and clinical studies should be performed to validate the proposed biomarkers and extend our findings to develop effective clinical strategies for diagnostic and therapeutic purposes.

## Data Availability

Publicly available datasets were analyzed in this study. The names of the repository/repositories and accession number(s) can be found in the article/Supplementary Material.

## References

[B1] Ahmadpour S. T. Maheo K. Servais S. Brisson L. Dumas J. F. (2020). Cardiolipin, the mitochondrial signature lipid: Implication in cancer. Int. J. Mol. Sci. 21, 8031. 10.3390/ijms21218031 PMC7662448 33126604

[B2] Arakaki A. K. S. Pan W. A. Trejo J. A. (2018). GPCRs in cancer: Protease-activated receptors, endocytic adaptors and signaling. Int. J. Mol. Sci. 19, 18866–E1924. 10.3390/ijms19071886 PMC6073120 29954076

[B3] Aydin B. Arga K. Y. (2019). Co-expression network analysis elucidated a core module in association with prognosis of nonfunctioning non-invasive human pituitary adenoma. Front. Endocrinol. 10, 361–415. 10.3389/fendo.2019.00361 PMC6563679 31244774

[B4] Bader G. D. Hogue C. W. V. (2003). An automated method for finding molecular complexes in large protein interaction networks. BMC Bioinforma. 4, 2. 10.1186/1471-2105-4-2 PMC149346 12525261

[B5] Bell D. W. (2014). Novel genetic targets in endometrial cancer. Expert Opin. Ther. Targets 18, 725–730. 10.1517/14728222.2014.909414 24750045 PMC4128487

[B6] Bersini S. Lytle N. K. Schulte R. Huang L. Wahl G. M. Hetzer M. W. (2020). Nup93 regulates breast tumor growth by modulating cell proliferation and actin cytoskeleton remodeling. Life Sci. Alliance 3, 2019006233–e201900714. 10.26508/LSA.201900623 PMC6971368 31959624

[B7] Beshnova D. A. Cherstvy A. G. Vainshtein Y. Teif V. B. (2014). Regulation of the nucleosome repeat length *in vivo* by the DNA sequence, protein concentrations and long-range interactions. PLoS Comput. Biol. 10 (7), e1003698. 10.1371/journal.pcbi.1003698 24992723 PMC4081033

[B8] Bolstad B. M. Irizarry R. A. Astrand M. Speed T. P. (2003). A comparison of normalization methods for high density oligonucleotide array data based on variance and bias. Bioinformatics 19, 185–193. 10.1093/bioinformatics/19.2.185 12538238

[B9] Bowen N. J. Walker L. D. Matyunina L. V. Logani S. Totten K. A. Benigno B. B. (2009). Gene expression profiling supports the hypothesis that human ovarian surface epithelia are multipotent and capable of serving as ovarian cancer initiating cells. BMC Med. Genomics 2, 71. 10.1186/1755-8794-2-71 20040092 PMC2806370

[B10] Cai C. Zhang Y. Hu X. Hu W. Yang S. Qui H. (2021). CDT1 is a novel prognostic and predictive biomarkers for hepatocellularcarcinoma. Front. Oncol. 11, 721644. 10.3389/fonc.2021.721644 34631549 PMC8497762

[B11] Chang L. Zhou G. Soufan O. Xia J. (2020). miRNet 2.0: Network-based visual analytics for miRNA functional analysis and systems biology. Nucleic Acids Res. 48, W244–W251. 10.1093/nar/gkaa467 32484539 PMC7319552

[B12] Chen S. N. Chang R. Lin L. Te Chern C. U. Tsai H. W. Wen Z. H. (2019). MicroRNA in ovarian cancer: Biology, pathogenesis, and therapeutic opportunities. Int. J. Environ. Res. Public Health 16, E1510–E1514. 10.3390/ijerph16091510 31035447 PMC6539609

[B13] Chen X. Yang H. Liu G. Zhang Y. (2021). Nucome: A comprehensive database of nucleosome organization referenced landscapes in mammalian genomes. BMC Bioinforma. 22, 321. 10.1186/s12859-021-04239-9 PMC8201709 34120586

[B14] Chin C. H. Chen S. H. Wu H. H. Ho C. W. Ko M. T. Lin C. Y. (2014). cytoHubba: identifying hub objects and sub-networks from complex interactome. BMC Syst. Biol. 8 (4), S11–S17. 10.1186/1752-0509-8-S4-S11 25521941 PMC4290687

[B15] Coscia F. Watters K. M. Curtis M. Eckert M. A. Chiang C. Y. Tyanova S. (2016). Integrative proteomic profiling of ovarian cancer cell lines reveals precursor cell associated proteins and functional status. Nat. Commun. 7, 12645. 10.1038/ncomms12645 27561551 PMC5007461

[B16] Curtis M. Kenny H. A. Ashcroft B. Mukherjee A. Johnson A. Zhang Y. (2019). Fibroblasts mobilize tumor cell glycogen to promote proliferation and metastasis. Cell. Metab. 29, 141–155. 10.1016/j.cmet.2018.08.007 30174305 PMC6326875

[B17] Didžiapetriene J. Bublevič J. Smailyte G. Kazbariene B. Stukas R. (2014). Significance of blood serum catalase activity and malondialdehyde level for survival prognosis of ovarian cancer patients. Medicina 50, 204–208. 10.1016/j.medici.2014.09.001 25458956

[B18] Du F. Li X. Feng W. Qiao C. Chen J. Jiang M. (2020). SOX13 promotes colorectal cancer metastasis by transactivating SNAI2 and c-MET. Oncogene 39, 3522–3540. 10.1038/s41388-020-1233-4 32111984

[B19] Egan K. Crowley D. Smyth P. O’Toole S. Spillane C. Martin C. (2011). Platelet adhesion and degranulation induce pro-survival and pro-angiogenic signalling in ovarian cancer cells. PLoS One 6, e26125. 10.1371/journal.pone.0026125 22022533 PMC3192146

[B20] Fasihi A. Soltani B. M. Atashi A. Nasiri S. (2018). Introduction of *hsa-miR-103a* and *hsa-miR-1827* and *hsa-miR-137* as new regulators of Wnt signaling pathway and their relation to colorectal carcinoma. J. Cell. Biochem. 119, 5104–5117. 10.1002/jcb.26357 28817181

[B21] Gov E. Arga K. Y. (2017). Differential co-expression analysis reveals a novel prognostic gene module in ovarian cancer. Sci. Rep. 7, 4996. 10.1038/s41598-017-05298-w 28694494 PMC5504034

[B22] Gov E. (2020). Co-expressed functional module-related genes in ovarian cancer stem cells represent novel prognostic biomarkers in ovarian cancer. Syst. Biol. Reprod. Med. 66, 255–266. 10.1080/19396368.2020.1759730 32441533

[B23] Halle M. K. Sødal M. Forsse D. Engerud H. Woie K. Lura N. G. (2021). A 10-gene prognostic signature points to LIMCH1 and HLA-DQB1 as important players in aggressive cervical cancer disease. Br. J. Cancer 124, 1690–1698. 10.1038/s41416-021-01305-0 33723390 PMC8110544

[B24] He L. Guo L. Vathipadiekal V. Sergent P. A. Growdon W. B. Engler D. A. (2014). Identification of LMX1B as a novel oncogene in human ovarian cancer. Oncogene 33, 4226–4235. 10.1038/onc.2013.375 24056967

[B25] Jia Y. Shi H. Cao Y. Feng W. Li M. Li X. (2019). PDZ and LIM domain protein 4 suppresses the growth and invasion of ovarian cancer cells via inactivation of STAT3 signaling. Life Sci. 233, 116715–116719. 10.1016/j.lfs.2019.116715 31376371

[B26] Kanehisa M. Goto S. (2000). Kegg: Kyoto encyclopedia of genes and genomes. Nucleic Acids Res. 28, 27–30. 10.1093/nar/28.1.27 10592173 PMC102409

[B27] Kanellou A. Giakoumakis N. N. Panagopoulos A. Tsaniras S. C. Lygerou Z. (2020). The licensing factor Cdt1 links cell cycle progression to the DNA damage response. Anticancer Res. 40, 2449–2456. 10.21873/anticanres.14214 32366388

[B28] Kori M. Gov E. Arga K. Y. (2019). Novel genomic biomarker candidates for cervical cancer as identified by differential co-expression network analysis. Omi. A J. Integr. Biol. 23, 261–273. 10.1089/omi.2019.0025 31038390

[B29] Lee W. J. Shin C. H. Ji H. Jeong S. D. Park M. S. Won H. H. (2021). hnRNPK-regulated LINC00263 promotes malignant phenotypes through miR-147a/CAPN2. Cell. Death Dis. 12, 290–318. 10.1038/s41419-021-03575-1 33731671 PMC7969774

[B30] Li Q. Liu S. Yan J. Sun M. Z. Greenaway F. T. (2021). The potential role of miR-124-3p in tumorigenesis and other related diseases. Mol. Biol. Rep. 48, 3579–3591. 10.1007/s11033-021-06347-4 33877528

[B31] Liao Y. Wang Y. Cheng M. Huang C. Fan X. (2020). Weighted gene coexpression network analysis of features that control cancer stem cells reveals prognostic biomarkers in lung adenocarcinoma. Front. Genet. 11, 311–314. 10.3389/fgene.2020.00311 32391047 PMC7192063

[B32] Lin L. Hu K. (2021). MİR-147: Functions and implications in inflammation and diseases. Microrna 10 (2), 91–96. 10.2174/2211536610666210707113605 34238178 PMC8714690

[B33] Lin Y. H. Zhen Y. Y. Chien K. Y. Lee I. C. Lin W. C. Chen M. Y. (2017). LIMCH1 regulates nonmuscle myosin-II activity and suppresses cell migration. Mol. Biol. Cell. 28, 1054–1065. 10.1091/mbc.E15-04-0218 28228547 PMC5391182

[B34] Liu Y. Tian F. Hu Z. Delisi C. (2015). Evaluation and integration of cancer gene classifiers: Identification and ranking of plausible drivers. Sci. Rep. 5, 10204–10215. 10.1038/srep10204 25961669 PMC4650817

[B35] Lloyd K. L. Cree I. A. Savage R. S. (2015). Prediction of resistance to chemotherapy in ovarian cancer: A systematic review. BMC Cancer 15, 117–132. 10.1186/s12885-015-1101-8 25886033 PMC4371880

[B36] Lu J. Huang X. Y. Wang Y. H. Xie J. W. Wang J. B. Lin J. X. (2020). POC1A acts as a promising prognostic biomarker associated with high tumor immune cell infiltration in gastric cancer. Aging 12, 18982–19011. 10.18632/aging.103624 33052878 PMC7732308

[B37] Lu Y. Luan X. R. (2019). miR-147a suppresses the metastasis of non-small-cell lung cancer by targeting CCL5. J. Int. Med. Res. 48, 300060519883098–14. 10.1177/0300060519883098 31884861 PMC7607764

[B38] Madden S. F. Clarke C. Stordal B. Carey M. S. Broaddus R. Gallagher W. M. (2014). OvMark: A user-friendly system for the identification of prognostic biomarkers in publically available ovarian cancer gene expression datasets. Mol. Cancer 13, 241–11. 10.1186/1476-4598-13-241 25344116 PMC4219121

[B39] Mao Y. Nie Q. Yang Y. Mao G. (2020). Identification of co-expression modules and hub genes of retinoblastoma via co-expression analysis and protein-protein interaction networks. Mol. Med. Rep. 22, 1155–1168. 10.3892/mmr.2020.11189 32468072 PMC7339782

[B40] Mishra S. Bernal C. Silvano M. Anand S. Altaba A. R. (2019). The protein secretion modulator TMED9 drives CNIH4/TGFα/GLI signaling opposing TMED3-WNT-TCF to promote colon cancer metastases. Oncogene 38, 5817–5837. 10.1038/s41388-019-0845-z 31253868 PMC6755966

[B41] Mok S. C. Bonome T. Vathipadiekal V. Bell A. Johnson M. E. Wong k. (2009). A gene signature predictive for outcome in advanced ovarian cancer identifies a survival factor: Microfibril-associated glycoprotein 2. Cancer Cell. 16, 521–532. 10.1016/j.ccr.2009.10.018 19962670 PMC3008560

[B42] Otálora-Otálora B. A. Florez M. López-Kleine L. Canas Arboleda A. Grajales Urrego D. M. Rojas A. (2019). Joint transcriptomic analysis of lung cancer and other lung diseases. Front. Genet. 10, 1260–18. 10.3389/fgene.2019.01260 31867044 PMC6908522

[B43] Pan W. W. Zhou J. J. Yu C. Xu Y. Guo L. J. Zhang H. Y. (2013). Ubiquitin E3 ligase CRL4CDT2/DCAF2 as a potential chemotherapeutic target for ovarian surface epithelial cancer. J. Biol. Chem. 288, 29680–29691. 10.1074/jbc.M113.495069 23995842 PMC3795265

[B44] Piñero J. Ramírez-Anguita J. M. Saüch-Pitarch J. Ronzano F. Centeno E. Sanz F. (2020). The DisGeNET knowledge platform for disease genomics: 2019 update. Nucleic Acids Res. 48, D845–D855. 10.1093/nar/gkz1021 31680165 PMC7145631

[B45] R Core Team (2020). A language and environment for statistical computing. Vienna, Austria: R Development Core Team. Available at: http://www.r-project.org/index.html .

[B46] Sauvageau E. Rochdi M. D. Oueslati M. Hamdan F. F. Percherancier Y. Simpson J. C. (2014). CNIH4 interacts with newly synthesized GPCR and controls their export from the endoplasmic reticulum. Traffic 15, 383–400. 10.1111/tra.12148 24405750

[B47] Shannon P. Markiel A. Ozier O. Baliga N. S. Wang J. T. Ramage D. (2003). Cytoscape : A software environment for integrated models of biomolecular interaction networks. Genome Res. 13, 2498–2504. 10.1101/gr.1239303 14597658 PMC403769

[B48] Smyth G. K. (2004). Linear models and empirical bayes methods for assessing differential expression in microarray experiments. Stat. Appl. Genet. Mol. Biol. 3, 1027. 10.2202/1544-6115.1027 16646809

[B49] Sun T. Bi F. Liu Z. Yang Q. (2020). SLC7A2 serves as a potential biomarker and therapeutic target for ovarian cancer. Aging (Albany. NY) 12, 13281–13296. 10.18632/aging.103433 32647070 PMC7377849

[B50] Sun W. Gui L. Zuo X. Zhang L. Zhou D. Duan X. (2016). Human epithelial-type ovarian tumour marker beta-2-microglobulin is regulated by the TGF-β signaling pathway. J. Transl. Med. 14, 75–13. 10.1186/s12967-016-0832-x 26983758 PMC4793749

[B51] Thorne L. S. Rochford G. Williams T. D. Southam A. D. Rodriguez-Blanco G. Dunn W. B. (2021). Cytoglobin protects cancer cells from apoptosis by regulation of mitochondrial cardiolipin. Sci. Rep. 11, 985–16. 10.1038/s41598-020-79830-w 33441751 PMC7806642

[B52] Tone A. A. Virtanen C. Shaw P. A. Brown T. J. (2011). Decreased progesterone receptor isoform expression in luteal phase fallopian tube epithelium and high-grade serous carcinoma. Endocr. Relat. Cancer 18, 221–234. 10.1530/ERC-10-0235 21263043 PMC3043379

[B53] Tong M. Yu C. Zhan D. Zhang M. Zhen B. Zhu W. (2019). Molecular subtyping of cancer and nomination of kinase candidates for inhibition with phosphoproteomics: Reanalysis of CPTAC ovarian cancer. EBioMedicine 40, 305–317. 10.1016/j.ebiom.2018.12.039 30594550 PMC6412074

[B54] Usman S. Khawer M. Rafique S. Naz Z. Saleem K. (2020). The current status of anti-GPCR drugs against different cancers. J. Pharm. Anal. 10, 517–521. 10.1016/j.jpha.2020.01.001 33425448 PMC7775845

[B55] Venkataramanan S. Mathavan S. (2020). Identification of potential biomarkers and their pathways for breast cancer using integrated bioinformatics analysis. Eur. J. Mol. Clin. Med. 7, 551–561.

[B56] Wang H. Huang H. Wang L. Liu Y. Wang M. Zhao S. (2021a). Cancer-associated fibroblasts secreted miR-103a-3p suppresses apoptosis and promotes cisplatin resistance in non-small cell lung cancer. Aging (Albany. NY) 13, 14456–14468. 10.18632/aging.103556 33999859 PMC8202839

[B57] Wang Z. Pan L. Guo D. Luo X. Tang J. Yang W. (2021b). A novel five-gene signature predicts overall survival of patients with hepatocellular carcinoma. Cancer Med. 10, 3808–3821. 10.1002/cam4.3900 33934539 PMC8178492

[B58] Wu M. Sun Y. Wu J. Liu G. (2020). Identification of hub genes in high-grade serous ovarian cancer using weighted gene co-expression network analysis. Med. Sci. Monit. 26, e922107–21. 10.12659/MSM.922107 32180586 PMC7101203

[B59] Wu X. Yu M. Zhang Z. Leng F. Ma Y. Xie N. (2021). DDB2 regulates DNA replication through PCNA-independent degradation of CDT2. Cell. Biosci. 11, 34–12. 10.1186/s13578-021-00540-5 33557942 PMC7869461

[B60] Xu Y. Gao W. Zhang Y. Wu S. Liu Y. Deng X. (2018). ABT737 reverses cisplatin resistance by targeting glucose metabolism of human ovarian cancer cells. Int. J. Oncol. 53, 1055–1068. 10.3892/ijo.2018.4476 30015875 PMC6065457

[B61] Yang Z. Y. Liu X. Y. Shu J. Zhang H. Ren Y. Q. Xu Z. B. (2019). Multi-view based integrative analysis of gene expression data for identifying biomarkers. Sci. Rep. 9, 13504–15. 10.1038/s41598-019-49967-4 31534156 PMC6751173

[B62] Yi Q. Wei J. Li Y. (2022). Effects of miR-103a-3p targeted regulation of TRIM66 Axis on docetaxel resistance and glycolysis in prostate cancer cells. Front. Genet. 12, 813793–12. 10.3389/fgene.2021.813793 35211152 PMC8861206

[B63] Zhang J. Li X. Liu X. Tian F. Zeng W. Xi X. (2018). EIF5A1 promotes epithelial ovarian cancer proliferation and progression. Biomed. Pharmacother. 100, 168–175. 10.1016/j.biopha.2018.02.016 29428664

[B64] Zhang Y. Zhang Y. Xu H. (2019). LIMCH1 suppress the growth of lung cancer by interacting with HUWE1 to sustain p53 stability. Gene 712, 143963. 10.1016/j.gene.2019.143963 31279706

[B65] Zhao Y. Wang J. Liang F. Liu Y. Wang Q. Zhang H. (2019). NucMap: A database of genome-wide nucleosome positioning map across species. Nucleic Acids Res. 47, D163–D169. 10.1093/nar/gky980 30335176 PMC6323900

[B66] Zhou Y. Zhou B. Pache L. Chang M. Khodabakhshi A. H. Tanaseichuk O. (2019). Metascape provides a biologist-oriented resource for the analysis of systems-level datasets. Nat. Commun. 10, 1523. 10.1038/s41467-019-09234-6 30944313 PMC6447622

[B67] Zhu F. J. Li J. Z. Wang L. L. (2020). MicroRNA-1-3p inhibits the growth and metastasis of ovarian cancer cells by targeting DYNLT3. Eur. Rev. Med. Pharmacol. Sci. 24, 8713–8721. 10.26355/eurrev_202009_22808 32964959

